# Present-day monitoring underestimates the risk of exposure to pathogenic bacteria from cold water storage tanks

**DOI:** 10.1371/journal.pone.0195635

**Published:** 2018-04-12

**Authors:** Aji Peter, Edwin Routledge

**Affiliations:** 1 Institute of Environment, Health and Societies, Brunel University London, Uxbridge, Middlesex, United Kingdom; 2 Aqua Technologies Europe Ltd, Hounslow, Middlesex, United Kingdom; University of Pittsburgh, UNITED STATES

## Abstract

Water-borne bacteria, found in cold water storage tanks, are causative agents for various human infections and diseases including Legionnaires’ disease. Consequently, regular microbiological monitoring of tank water is undertaken as part of the regulatory framework used to control pathogenic bacteria. A key assumption is that a small volume of water taken from under the ball valve (where there is easy access to the stored water) will be representative of the entire tank. To test the reliability of this measure, domestic water samples taken from different locations of selected tanks in London properties between November 2015 and July 2016 were analysed for TVCs, Pseudomonas and Legionella at an accredited laboratory, according to regulatory requirements. Out of ~6000 tanks surveyed, only 15 were selected based on the ability to take a water sample from the normal sampling hatch (located above the ball valve) and from the far end of the tank (usually requiring disassembly of the tank lid with risk of structural damage), and permission being granted by the site manager to undertake the additional investigation and sampling. Despite seasonal differences in water temperature, we found 100% compliance at the ball valve end. In contrast, 40% of the tanks exceeded the regulatory threshold for temperature at the far end of the tank in the summer months. Consequently, 20% of the tanks surveyed failed to trigger appropriate regulatory action based on microbiological analyses of the water sample taken under the ball valve compared to the far end sample using present-day standards. These data show that typical water samples collected for routine monitoring may often underestimate the microbiological status of the water entering the building, thereby increasing the risk of exposure to water bourne pathogens with potential public health implications. We propose that water storage tanks should be redesigned to allow access to the far end of tanks for routine monitoring purposes, and that water samples used to ascertain the regulatory compliance of stored water in tanks should be taken at the point at which water is abstracted for use in the building.

## Introduction

Potable water is typically produced at water treatment facilities where incoming water is treated to remove pathogens and is disinfected before it leaves the treatment works [1]. A small residual amount of chlorine is left in the water to maintain quality as it travels through the network of mains and pipes that deliver this water to various residences. Despite this, treated water can become contaminated with microorganisms during transportation throughout the pipework network, and during storage [2]. Long horizontal installations of pipework, the types of materials used for the pipework and fittings, deadlegs (isolated sections of piping) and excessive water storage or stagnation can all affect water quality and encourage the proliferation of many species of bacteria [3]. In order to protect society against the harmful effects of exposure to pathogenic bacteria, many countries throughout the world have developed and adopted standards used for the evaluation of microbiological status of point-of-use and point-of-entry potable water in buildings. The United States Environmental Protection Agency (USEPA) and the European Environment Agency (under the EU Water Framework Directive) have implemented monitoring and sampling strategies to ensure that the health of building occupants is protected from unabated proliferation of pathogenic bacteria [4,5]. In the UK, all water samples taken for microbiological assessment are taken, transported and analysed under UKAS accredited conditions as stipulated by drinking water inspectrate (DWI) for compliance [6].

**Fig 1:**
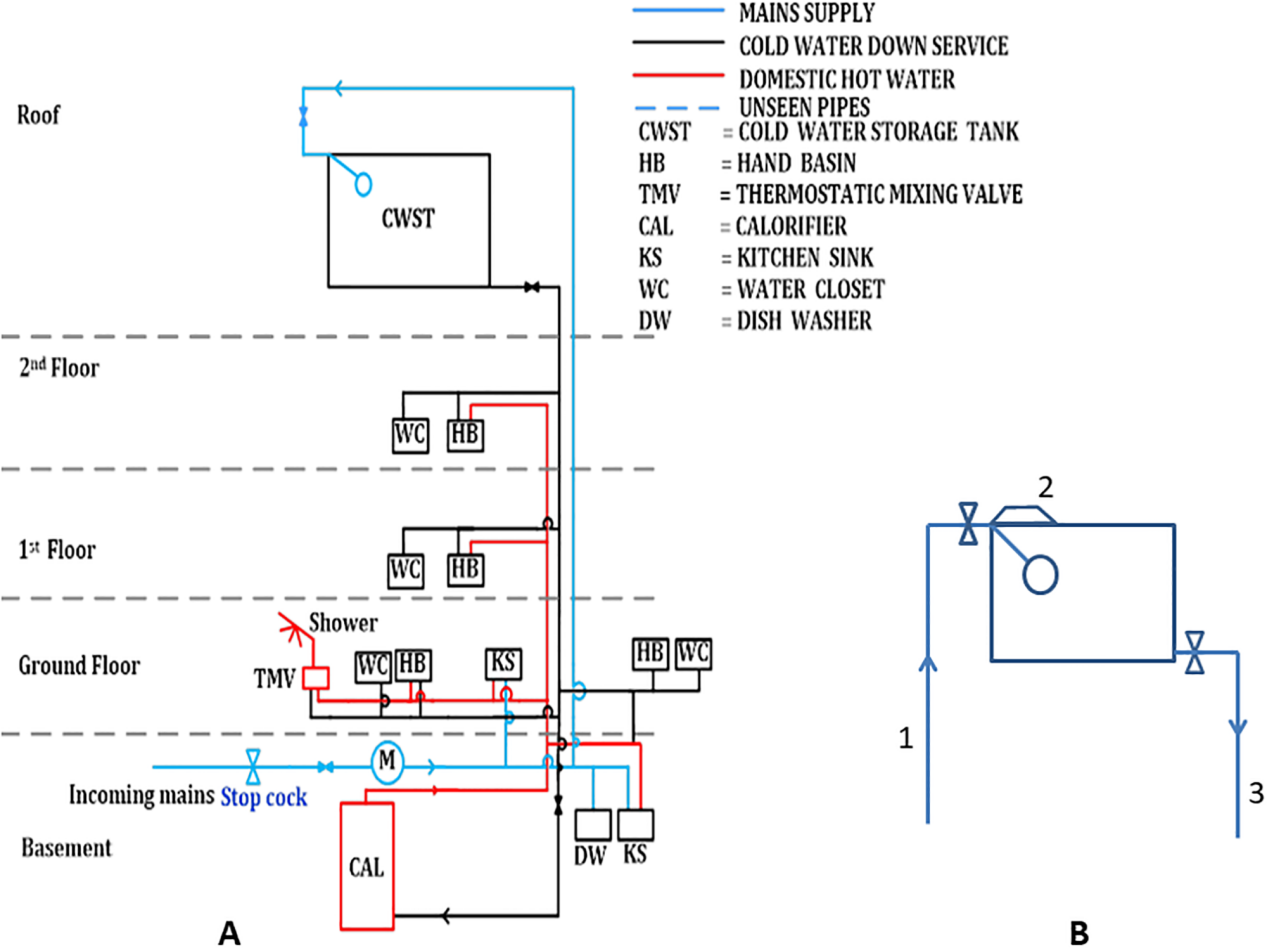
Schematic of a typical water system. A) Schematic of typical gravity-fed water system found in may commercial and older reseidntial buildings. B) Schematic diagram of a typical cold water storage tank as per WRAS Guidelines 1. Incoming mains with isolation valve connected to ball valve; 2. Inspection acces hatch situated above the ball valve to assist with maintenance; 3. Outlet of the tank with isolation valve.

Cold water storage tanks are one of the most important elements of concern, being both the point-of-entry of potable water into buildings, and the reservoir of water used to supply the entire building [7]. The majority of the older properties in the UK have cold water storage tanks located in the loft space or on the roof. These tanks usually feed cold water taps by gravity (with the exception of the kitchen cold water tap) and the hot water calorifiers [8] (Fig 1A). In modern buildings, cold water storage tanks are located in either the basement or on the ground floor of the building. These tanks are connected to booster pump sets to provide stored water to the entire building. The internal condition of these storage tanks has a direct impact on the quality of stored water, even if the tanks are properly designed, correctly installed and kept in good external order [8]. Factors such as the tank construction method, the materials used, plumbing arrangements, internal water flow and tank location (the ambient temperature around the tank) have a direct impact on the internal environment of the tank, and conditions may arise that encourage the proliferation of pathogenic bacteria, including *Legionella* [9, 10]. Mains water is known to contain a variety of minerals (often referred to as hardness), and can contain relatively high concentrations of calcium and magnesium [11] that contribute to the formation of scale which adheres firmly to internal surfaces of water storage tanks [12]. Moreover, tiny suspended solids and dissolved solids in the mains water settle and collect at the bottom of the tank as sediments. In the case of metal tanks, electrochemical corrosion results in the formation of stable metal oxides (rust) that can remain within the water tank for a long time [13]. Scale, sediments and corrosion products serve as nutrients, that encourage *Legionella* proliferation [14]. The plumbing arrangement will also affect the internal environment of the tank [15]. If the inlet (incoming mains) and outlets are on the same side, then internal water circulation may be hindered leading to greater water stagnation within the tank. The combination of an oversized mains inlet pipe and a relatively small outlet can also lead to water stagnation within the water storage tank [16], thereby increasing the temperature of stored water and contributing to biofilm formation; a perfect breeding ground for *Legionella* bacteria [17, 18]. In order to ensure that water quality standards are met, and to maintain a healthy water system, routine water tank inspections and stored water sample analysis is necessary [19]. Analysis of ‘representative water samples’ collected from any water system are an important tool in the armoury used to evaluate the human health risk posed by a condition of a particular water system [20]. According to Water Regulations Advisory Scheme (WRAS), the water storage tank should have an access hatch above the mains inlet valve of the storage tank (Fig 1B) to enable routine internal inspection and water sampling [21].

The key assumption used in currect practice is that a small volume of water taken from under the ball valve (where there is easy access to the stored water) will be representative of the entire tank. A representative sample is a small quantity of something whose characteristics represent (as accurately as possible) the entire batch [22]. Obtaining a representative sample is the most important factor for a relevant description of the environment [23], especially when the result will be used for regulatory purposes and to protext public health. According to The Drinking Water Inspectorate (DWI), ‘samples must be taken from locations that are representative of the water source, storage facilities, distribution network and points at which water is delivered to the consumer. These points should include those that yield samples representative of the conditions at the most unfavourable sources or places in the supply system, particularly points of possible contamination such as unprotected sources, loops, reservoirs, low-pressure zones, ends of the system, etc’. [20]. Here we explore the possible limitations of assessing the actual risk factors within the water tank during present-day routine visual inspection and collection of a ‘representative sample’ used for microbiological analysis according to DWI.

## Methods

### Tank selection

Service engineers contracted for routine inspection and maintenance by a specialised water hygiene management company (Aqua Technologies Europe Ltd) were used to identify suitable tanks to include in the study. According to the Drinking Water Inspectorate (DWI) standards, cold water storage tanks must be completely sealed with the exception of one access point for inspection, monitoring and maintenance situated above the ball valve. Therefore, selection criteria for suitable tanks for inclusion in the study was based on (i) the ability to take a water sample from the normal sampling hatch (located above the ball valve) and from the far end of the tank (usually requiring disassembly of the tank lid with risk of structural damage), and (ii) permission being granted by the site manager to undertake the additional investigation and sampling. Out of approximately 6000 coldwater tanks surveyed over a 12 month period (July 2015-July 2016) only 15 suitable cold water storage tanks meeting the sample access criteria were identified by service engineers at various sites located in different London Boroughs. Permission was granted by site managers to gain access the far end of the tank and carry out the additional inspection work in every case.

### Tank inspection

Surveyed tanks were constructed from various materials, incuding metal tanks (galvanised iron), metal tanks with internal butyl lining, fibreglass tanks, plastic tanks and modern GRP (glass reinforced plastic) tanks. The external dimensions of each tank was measured in metres using a standard tape measure in order to calculate the capacity of each tank. The location of each tank in the building was recorded together with the inspection and sampling date (Table 1). Temperature of (i) the mains water through inlet discharge (ball valve), (ii) the stored water just below the ball valve and (iii) of stored water at the far end of the tanks were also recorded using a Testo 925 Aktionsset Sensor type K digital thermometer (temperature range -50 up to +300°C). Internal visual inspection was carried out for all fifteen tanks; sedimentation level, presence of biofilm, presence of scale and corrosion level was recorded qualitatively as ‘negligible’, ‘slight’, ‘moderate’ and ‘heavy’, and these findings were converted into numerical data (Table 2). Tanks could only be accessed and inspected on one occasion as part of the routine service contract in place.

**Table 1:** Tank deatails.

Tank Reference	Dimensions (Metres)	Volume (M^3^)	Material	position	Sample Date
**T1**	3.0 x 1.5 x 1.2	5.4	Metal	Roof	Nov 15
**T2**	2.5 x 1.4 x 1.35	4.7	Metal	Roof	Dec 15
**T3**	4.0 x 2.0 x 2.0	16.0	GRP	Ground floor	Dec 15
**T4**	2.5 x 1.3 x 1.2	3.9	Fibreglass	Roof	Jan 16
**T5**	2.7 x 1.6 x 1.5	6.5	Metal	Roof	Jan 16
**T6**	6.0 x 2.0 x 3.0	36.0	GRP	Basement	Feb 16
**T7**	6.0 x 2.0 x 3.0	36.0	GRP	Basement	Feb 16
**T8**	1.9 x 0.8 x 0.8	1.2	Plastic	Roof	Mar 16
**T9**	3.2 x 2.0 x 1.5	9.6	Metal	Roof	Mar 16
**T10**	5.0 x 1.0 x 1.0	5.0	GRP	Ground floor	Apr 16
**T11**	4.0 x 4.0 x 2.0	32.0	GRP	Ground floor	Jun 16
**T12**	4.0 x 4.0 x 2.0	32.0	GRP	Ground floor	Jun 16
**T13**	4.0 x 1.7 x 1.5	10.2	Metal with butyl lining	Roof	July 16
**T14**	3.0 x 1.5 x 1.5	6.8	GRP	Basement	July 16
**T15**	1.0 x 0.7 x 0.8	0.6	Plastic	Roof	July 16

Details of the fifteen tanks assessed, including tank dimensions, capacity, construction material, position and sampling date.

**Table 2:** Relative scoring.

Tank Reference	Sedimentation		Biofilm		Scale		Corrosion	
	UBV	FE	UBV	FE	UBV	FE	UBV	FE
**T1**	0	1	0	1	1	1	1	1
**T2**	0	2	0	2	1	1	1	1
**T3 (P)**	0	1	0	1	0	0	0	0
**T4**	0	1	0	1	0	0	0	0
**T5**	1	2	0	2	0	0	1	1
**T6 (P)**	1	2	0	1	0	0	0	0
**T7 (P)**	1	2	0	1	0	0	0	0
**T8**	2	3	1	2	1	1	0	0
**T9**	2	3	1	3	1	1	1	2
**T10 (P)**	2	3	1	3	1	1	0	0
**T11 (P)**	1	3	0	1	0	0	0	0
**T12 (P)**	1	3	0	2	0	0	0	0
**T13**	1	3	1	3	1	1	0	0
**T14 (P)**	1	2	0	1	1	1	0	0
**T15**	0	0	0	0	0	0	0	0

Relative scoring of sedimentation, biofilm, scale and corrosion levels in each tank, where negligible (not visible) = 0; Slight = 1; Moderate = 2; Heavy = 3 (P) shows that the tank is designated for potable water use. With few exceptions, the level of sedimentation and biofilm increased in quantity/severity between UBV and FE. Also, this occurred in both cold water and potable water storage tanks.

### Water sample collection

Three water samples were collected from each of the tanks: one from the incoming mains (tank inlet), one from the tank just below the inlet ball valve (where routine sampling for pathogenic bacteria happens in practice), and one from the far end of the same tank. The samples were collected in sterile bottles (500ml, supplied by the UKAS accredited laboratory) in accordance with BS 8550:2010 guidelines for the collection of water samples. Water samples were stored in temperature controlled bags, protected from heat sources and sunlight, during transportation to the laboratory.

### Microbiological analysis

Water samples were analysed using standard UKAS protocols for TVC (3 days @ 22°C), TVC (2 days @ 37°C), *Pseudomonas*, *Escherichia coli* (E.coli), Coliforms and *Legionella pneumophila* in a UKAS accredited laboratory (Alcontrol Laboratories, UK) under the same laboratory conditions within 12 hours of collection. All the samples were collected identically and analysed by the same UKAS accredited laboratory to ensure the consistency and accuracy of the results produced. Assay detection limits were 1 cfu/ml (TVC—2 days at 37 °C, 3days at 22 °C and Pseudomonas) and 100 cfu/L (*Legionella*), based on UKAS accredited methods [24].

### Data analysis and interpretation

Absolute values (in cfu/volume of water collected) reported by accredited laboratories (data in S1 Table) are used to determine if remedial action is necessary. These values are never questioned for their precision or reliability. The entire regulatory system is based on ‘threshold’ levels, that once exceeded instigate regulatory action through non-compliance with the standards. Results of any repeated tests by accredited laboratories as part of their sample analysis processes are not reported, and any measures of variability (such as SD) are also not reported. The crucial research question highlighted here is therefore not whether two samples taken at different ends of the tank are different (from a statistical standpoint), but whether samples taken at different locations in the same tank (e.g. the ball valve end where samples are routinely taken) result in different regulatory actions compared to samples taken at a different location (e.g. the far end of the tank, where water is abstracted into the building). For this reason, absolute values from the accredited laboratory were used to determine if samples taken at different locations inside the tank were equivalent in terms of their compliance with regulatory thresholds.

#### Statistics

A Shapiro-Wilk test was conducted to confirm that TVCs, Pseudomonas and Legionella bacteria data in the UBV and FE samples were not normally distributed. A Wilcoxon signed rank test (which does not assume multivariate normality or homogeneity of variance) was then chosen. The null hypothesis was that the median difference between pairs of observations is zero for TVCs, Pseudomonas and Legionella bacteria reported in samples taken under the ball valve and from the far end of each tank.

## Results

**Fig 2:**
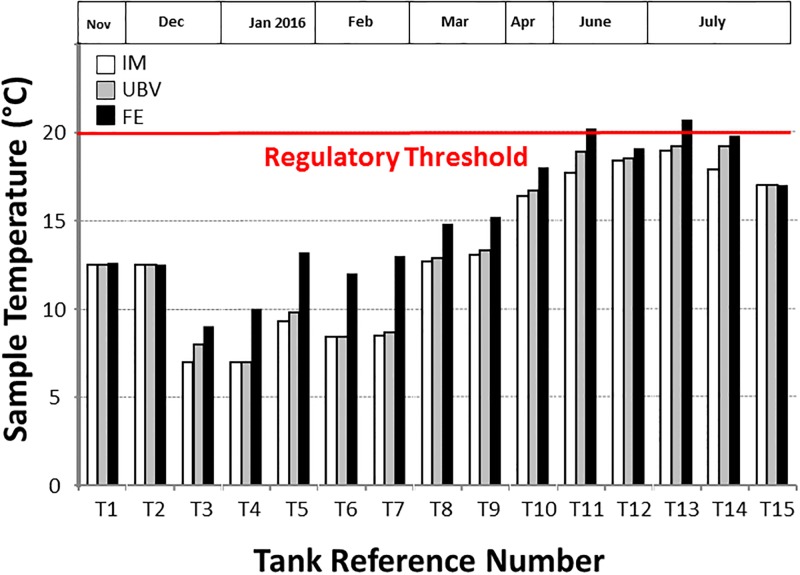
Sample temperature comparison. Sample temperature (°C) of the incoming mains water (IM), under the ball valve (UBV) and at the far end (FE) of fifteen operational cold water and potable water storage tanks in different London Boroughs recorded during routine inspection and sampling between November 2015 and July 2016. The red line shows the regulatory threshold of 20°C (acceptable limit) used for routine monitoring.

Fig 2 shows the temperature of the incoming mains (IM) water and storage water under the ball valve (UBV) and at the far end (FE) of fifteen tanks, recorded during routine inspection and sampling between November 2015 and July 2016. Seven tanks were situated on the ground floor or basement, and were connected to booster pumps to distribute potable water to the entire building. Eight tanks were located on the roof, with the purpose of distributing stored water to the calorifier(s) and cold water taps (with the exception of the kitchen tap). Storage water temperature varied with seasonality as expected, with water UBV temperatures as low as 7°C in December (winter time) rising to 19.2°C in July (British summer time). In all fifteen tanks, IM water temperature varied between 7–19°C (depending on the season), which was below the regulatory threshold of 20°C for both mains water and stored water [25]. The stored water increased in temperature by 0.1°C to 3.6°C from UBV to FE in the majority of tanks. In ten tanks, this temperature difference was >1°C, and in 4 tanks the temperature difference was > 3°C. The smallest water temperature differences (0°C to 0.1°C) occured in tanks below 6m^3^ in either the winter (November/December) or summer (July), whereas the greatest temperature gradient differences occurred in large tanks (36m^3^) sampled in February. Although UBV water temperatures were always below the regulatory threshold, the FE of Tank 14 reached the regulatory threshold limit (20°C), and Tanks 11 and 13 exceeded the regulatory threshold (20.2 and 20.7°C, respectively).

**Fig 3:**
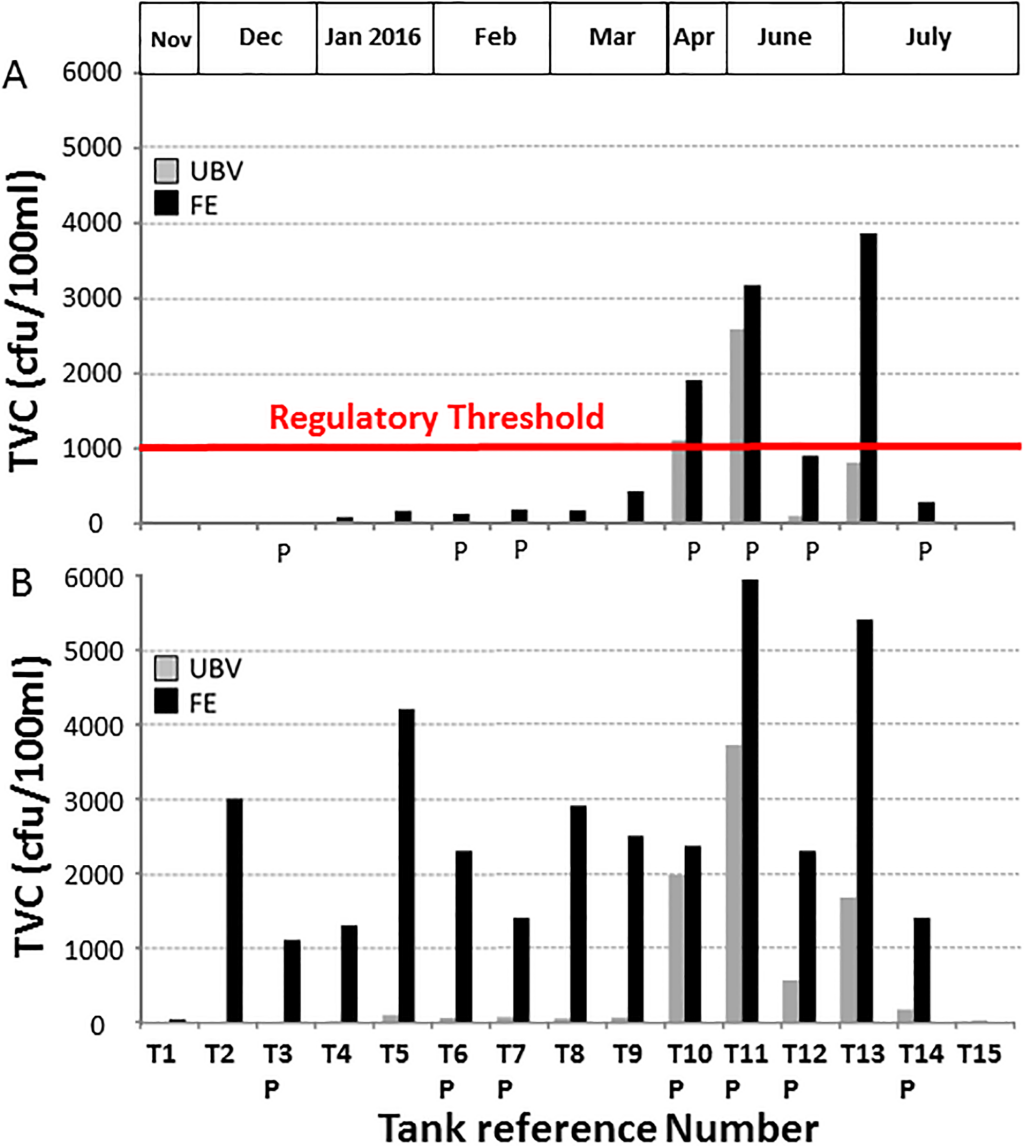
TVC analysis result comparison. Comparison of Total Viable Counts (expressed as colony forming units/100 ml) from water samples taken under the ball valve (UBV) and at the far end (FE) of fifteen independent cold water and potable water storage tanks located in different London Boroughs, and incubated at A) 37 °C for 2 days and B) 22 °C for 3 days. Tanks were sampled between November 2015 and July 2016. ‘P’ denotes tanks designated for potable water storage. The red line shows the regulatory threshold for TVC in potable water tanks from samples incubated at 37 °C for 2 days.

Fig 3 shows the water sample analysis results for Total Viable Counts (TVC) incubated for 3 days at 22°C (Fig 3A) and for 2 days at 37°C (Fig 3B), respectively. The TVC is an estimate of the total number of viable individual micro-organisms (including bacteria, fungi and mould species) present in a set volume of sample, and provides a relatively rapid quantitative insight of the microbiological status of the sample. Fourteen of the tanks sampled showed increases in TVC between samples taken UBV and FE and incubated for 3 days at 22°C, with the exception of Tank 15 that had 10 cfu/100ml at the both ends (Fig 3B). The biggest differences were observed in T2 and 3, where no TVCs were reported in the UBV sample, but 3000 and 1100 cfu/100ml were measured in the FE sample. T4 yielded 9 cfu/100ml in the UBV sample and 1300 cfu/100ml at the FE, producing in 144-fold difference (2 orders of magnitude) in TVCs between the UBV sample and the FE sample. The tanks sampled between November and March (with the exception of T4) showed a 40.2-fold (± 12.1 sd) mean increase in TVCs at the FE compared to UBV, and the tanks sampled in the summer had relatively higher TVCs in the UBV samples resulting in a 3.7-fold (± 2.5 sd) mean overall increase in TVCs at the FE. Thirteen out of fifteen tanks showed increased TVCs in FE samples compared to UBV samples incubated for 2 days at 37°C, and there were statistically greater TVC in FE samples collected from cold water storage tanks relative to their corresponding UBV samples (p = 0.0002).

UBV samples of potable water incubated for 2 days at 37°C were found to exceed the regulatory threshold of 1000 cfu/100ml on only two occasions (T10 and T11; Fig 3A). Fig 3A shows that the incubation temperature of 37°C favoured growth of microorganisms in water samples collected in April and into the summer. In T10 (sampled April 2016) the regulatory threshold for potable water was only just exceeded with the UBV sample yielding 1141 cfu/100ml, whereas the FE sample (1900 cfu/100ml) was clearly above the regulatory threshold.

**Fig 4:**
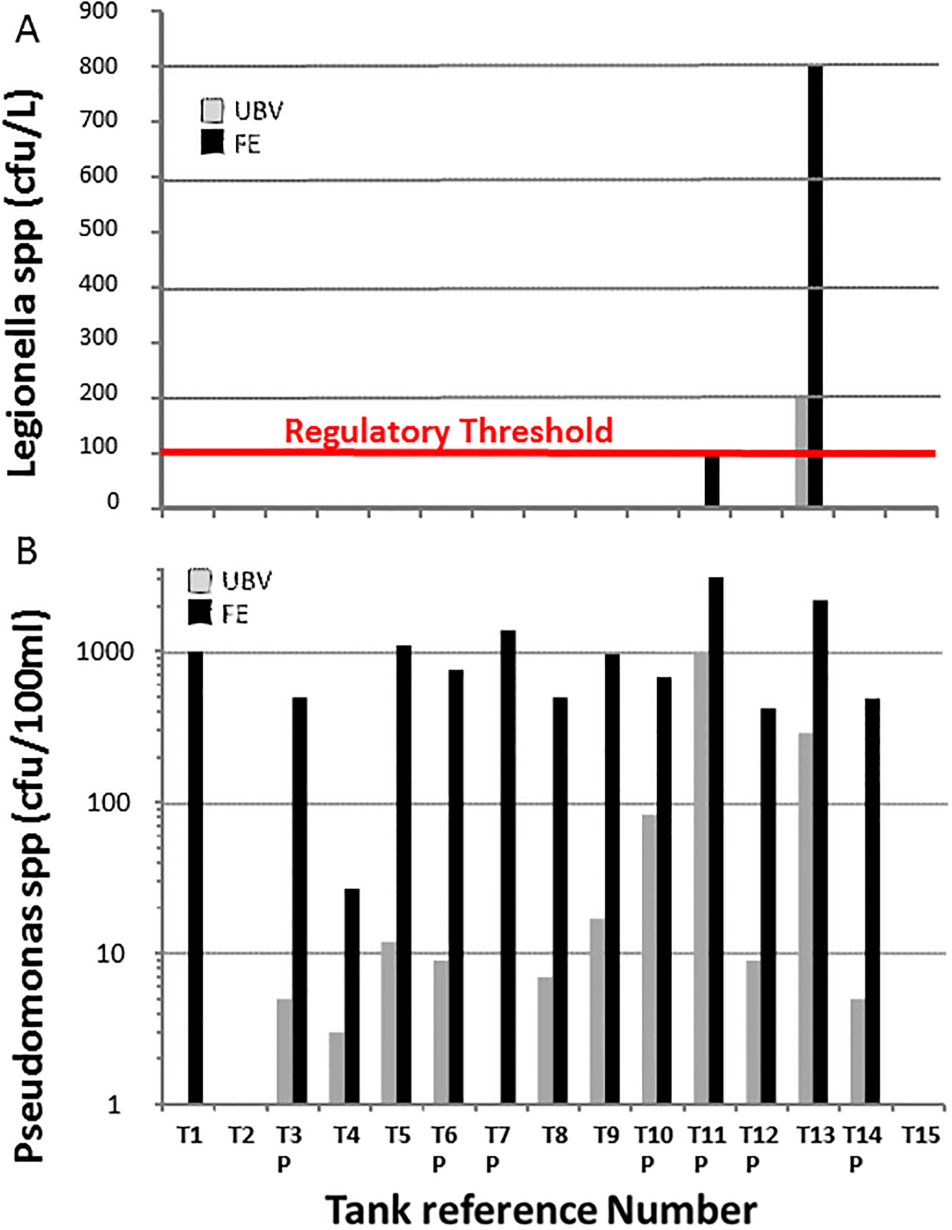
Comparison of Legionella and Pseudomonas analysis results. Comparison of (A) *Legionella* species (expressed as colon forming units/litre) and (B) Pseudomonas species (expressed as colony forming units/100ml) in water samples taken under the ball valve (UBV) and at the far end (FE) of fifteen independent cold water and potable water storage tanks located in different London Boroughs. Samples were taken between November 2015 and July 2016. ‘P’ denotes tanks that are designated for potable water use. The regulatory threshold for Pseudomonas in potable water is zero (line not shown) and is 100 cfu/L for *Legionella* in tank water.

Fig 4 shows the results of analysis of the water samples for both Pseudomonas and *Legionella* species. The regulatory threshold for Pseudomonas in potable water is 0 cfu/100ml and for *Legionella* in tank water it is 100 cfu/L. Fig 4a shows that most samples tested negative for *Legionella* in either UBV or FE samples, and there was a no statistical difference (p = 0.5) in Legionella bacteria counts found in FE samples collected from cold water storage tanks relative to their corresponding UBV sample. However, Tank 10 reached the regulatory threshold for *Legionella* (100 cfu/L) at the FE of the tank whereas *Legionella* was undetected under the ball valve. T12 also tested positive for *Legionella* in both UBV and FE samples, although the number of bacteria was 4-fold higher (800 cfu/L FE c.f. 200 cfu/L BVE) at the far end of the tank. Pseudomonas was regularly detected in tank water both in UBV and FE samples, with the exception of T2 and T15. In T7 (potable water) no Pseudomonas were detected in the UBV sample, whereas the FE sample yielded in excess of 1000 cfu/100ml thus exceeding the regulatory threshold. A similar finding occurred in T1, although this tank was not designated for potable water use. The UBV samples followed a seasonal trend, increasing from 7 cfu/100ml in March to a maximum of 980 cfu/100ml in June. In all tanks where Pseudomonas was detected in both UBV and FE samples, the number of bacteria in the FE sample was on average 54-fold higher (±39) and varied between 9-fold and 98-fold depending on the individual tank and the season of sampling. There were statistically greater Pseudomonas bacteria counts (p = 0.0002) in FE samples collected from cold water storage tanks relative to their corresponding UBV sample.

**Fig 5:**
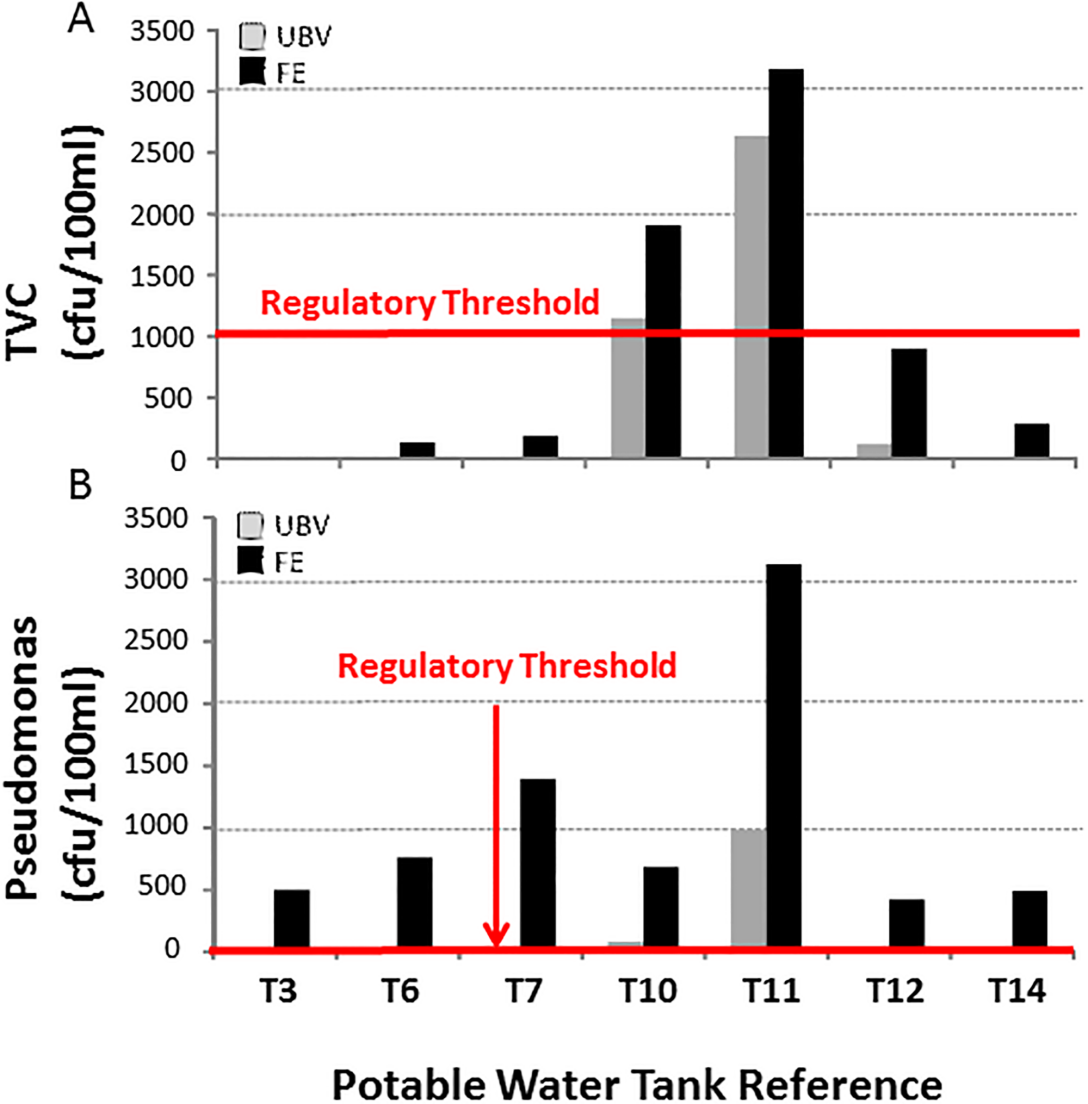
Comparison of TVC and Pseudomonas analysis results. Comparison of A) Total Viable Counts (TVC; 2 days incubation at 37°C) and B) Pseudomonas species (expressed as colony forming units/100ml) measured in water samples taken under the ball valve (UBV) and at the far end (FE) of potable water storage tanks located in different London Boroughs. Samples were taken between December 2015 and July 2016. The regulatory threshold (10 cfu/ml TVC and 0 cfu/ml Pseudomonas) is depicted using a red line.

Fig 5 shows a comparison of TVC and Pseudomonas species quantified in samples collected from both UBV and FE locations in potable water tanks at different times of the year. In all cases the UBV and FE samples provided a consistent course of action with respect to the regulatory threshold for TVCs incubated for 2 days at 37°C (10 cfu/ml), although T10 only just exceeded the regulatory threshold in the UBV sample (11 cfu/ml) compared to the FE sample (19 cfu/ml). In addition, in T12 the FE sample was approaching the regulatory threshold (9 cfu/ml) whereas the UBV sample was substantially lower (1 cfu/ml). In contrast, the levels of Pseudomonas in UBV samples was typically low (between 5 and 9 cfu/100ml) with the exception of T10 and T11 (83 and 980 cfu/100ml, respectively). All UBV samples, with the exception of T7, exceeded the regulatory thresdhold (0 cfu/ml). However, T7 exceeded the regulatory threshold in the FE sample (1390 cfu/100ml). There were consistently higher Pseudomonas counts in the FE sample relative to the UBV sample.

**Fig 6:**
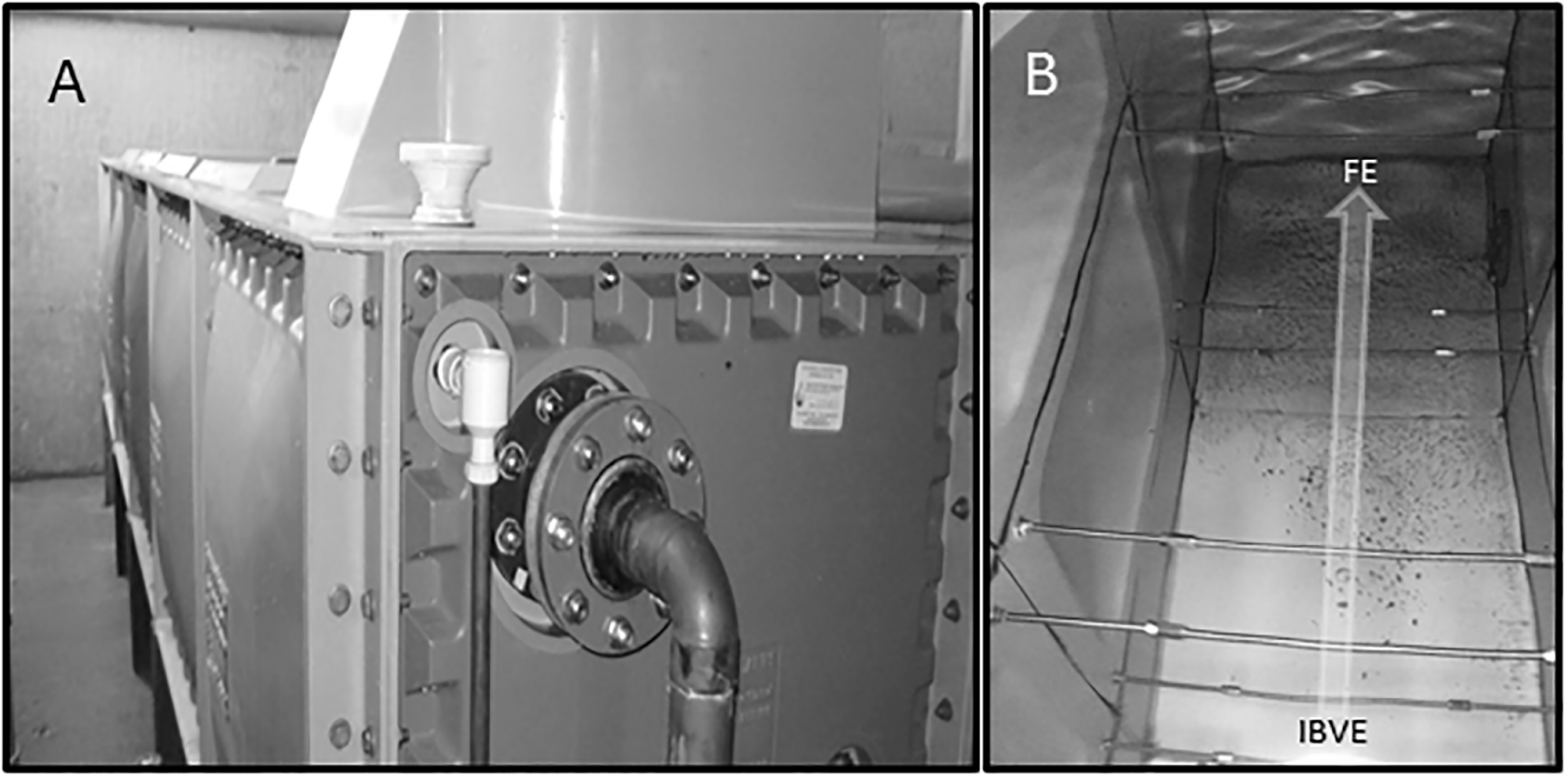
Long cold water storage tank. (A) Example of a 5 metre long GRP potable cold water storage tank showing the mains water inlet. (B) Sedimentation levels increase towards the far end (FE) of the tank from the inlet ball valve end (IBVE). The arrow shows the direction of mains water flow into the tank. Tank reference T10.

**Fig 7:**
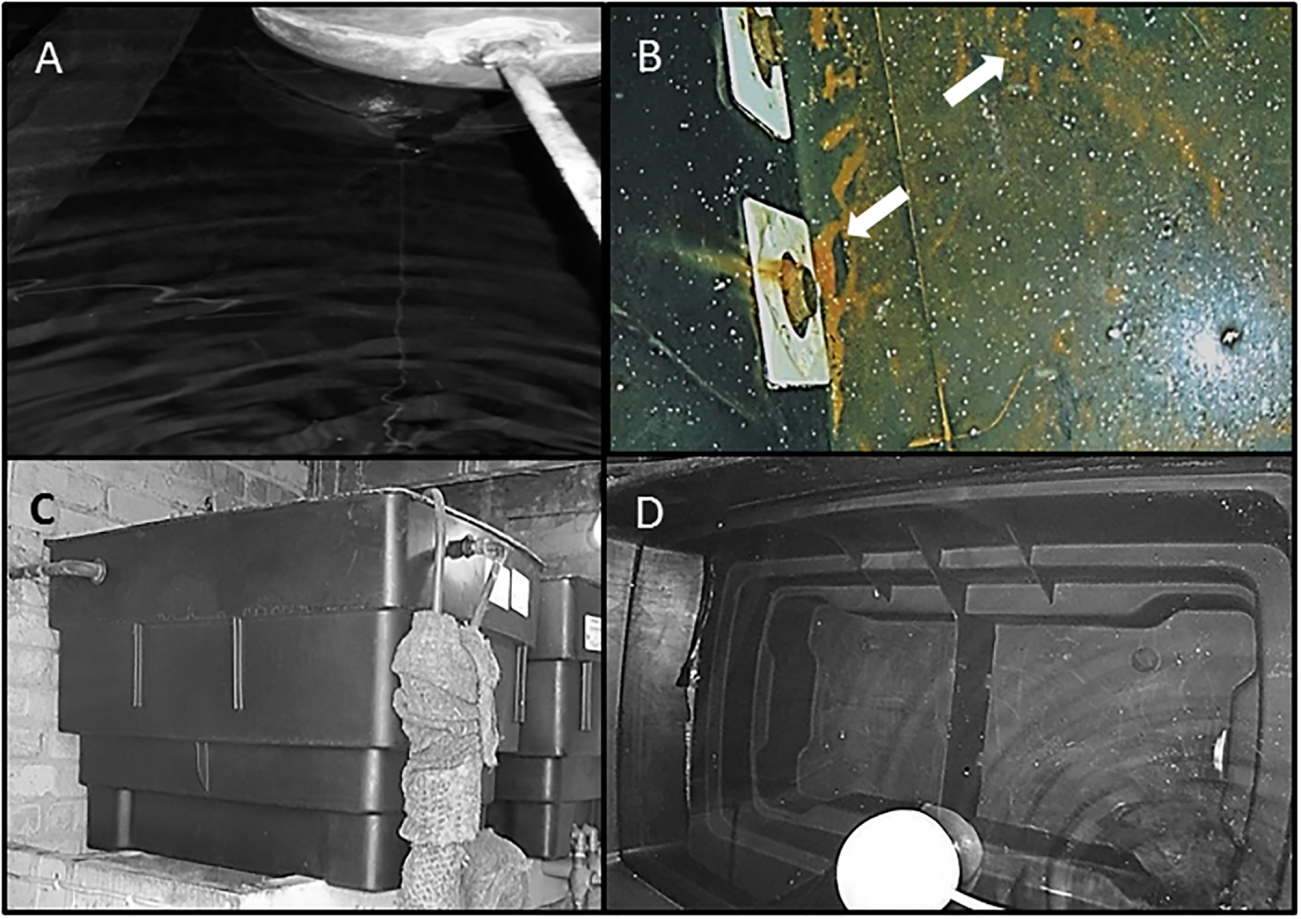
Comparison of long metal tank and small plastic tank. (A) 4 metre long metal cold water storage tank with butyl lining (T13) showing visibly clear stored water under the ball valve (A), compared to sediments and biofilm at the far end (B). (C, D) shows a 1 metre long plastic tank with visibly clear stored water (T15).

Visual inspection of the tanks showed a clear increase in the level of sedimentation with distance from the ball valve (see Figs 6 and 7), and presence of biofilm was also noted towards the far end of the tanks whereas under the ball valve, water appeared visibly clear (Fig 7A and 7B). The presence of a slight scale was noted in T1,T2,T8,T9,T10,T13 and T14, although it appeared to be similar at both ends of the tanks. There was evidence of slight corrosion throughout all the metal tanks, whereas in T9 the far end appeared as moderate (see Table 2). In the case of one metre long plastic tank (T15), water was apparently clear without sedimentation, stagnation, scale and biofilm (Fig 7D), and a temperature of 17°C was recorded for the incoming mains temperature and for the UBV and FE samples.

## Discussion

The microbiological analysis of water samples taken from a limited number of tanks throughout a year shows that identical regulatory actions (i.e. action or no action) would have occured in 80% of instances (based on data for TVCs, Pseudomonas and *Legionella* in each tank) irrespective of where the sample was taken, despite consistently higher numbers of bacteria at the far end of the tank. However, 3 out of 15 tanks surveyed failed to trigger appropriate regulatory action based on microbiological analyses of the water sample taken under the ball valve (n = 15 tanks) compared to the far end sample using present-day standards (see Table 3). Tanks that failed to trigger appropriate regulatory action were all sampled in late Spring and Summer, suggesting warming temperatures to be an important factor in this response. Indeed, tanks 11 and 13 both exceeded the threshold for temperature at the far end of the tank, despite being complaint at the ball valve end. The variation in temperature of the incoming mains water was due to seasonal changes, whilst differences between UBV and FE samples were due principally to water stagnation towards the far end of the tanks [26].

**Table 3:** Comparison of TVCs, Pseudomonas and *Legionella* (cfu/volume) from UBV and FE samples.

Tank	TVCs			Pseudomonas			*Legionella*		
	UBV (cfu/100ml)	FE (cfu/100 ml)	RT	UBV	FE	RT	UBV	FE	RT
7 (P)	1	185	1000 cfu/100ml	0	1390	0 cfu/ml	0	0	100 cfu/L
11(P)	2620	3170	1000 cfu/100ml	980	3110	0 cfu/ml	0	100	100 cfu/L
13	840	3850	1x 10^6^ cfu/100ml	290	2200	Not Checked	200	800	100 cfu/L

Unshaded boxes show agreement for ‘no-action’ (counts below regulatory threshold)

Orange boxes show agrrement for ‘action’ (counts above regulatory threshold).

Boxes in dark red show instances where there is a disagreement in compliance from UBV and FE samples

(P) denotes that the water was used for potable use.

RT denotes the regulatory threshold.

Most water contains microorganisms, and an estimation of their overall numbers provides important information used for system surveillance and water quality maintenance [27]. The Total Viable Count (TVC) is essentially a simple enumeration of all viable bacteria present in water [28]. Microorganisms growing better in laboratory media at 22°C reflect environmental micro-organisms and can be used to plot seasonal variations. In contrast, microorganisms that grow at 37°C may represent those of faecal origin [29]. TVC analysis (3 days incubation at 22°C) of incoming mains samples taken from T6, T9, T12 and T13 (1, 2, 12 and 10cfu/100ml, respectively; data not shown) were below the HSE’s Potable Water Standard limit of 100cfu/ml [30]. Also, TVC analysis (2 days incubation at 37°C) of mains water was non-detectable and below the regulatory threshold of 10cfu/ml [30]. Although the mains water and UBV temperatures recorded were almost identical, the temperatures recorded at the far end of the tanks were comparatively higher (Fig 2) thereby encouraging bacterial growth at the far end of most tanks [31]. According to a study carried out in USA, it was reported that bacteria in drinking water pose a health risk to all individuals, and especially patients with underlying health issues [28]. TVC analysis results (2 days at 37°C) for UBV and FE samples from T11p were approximately three times greater than the regulatory threshold of 1000cfu/100ml (2620 and 3170, respectively) raising serious concerns about the potable water quality in this building [30]. Despite this, we should note that regulatory action would have resulted from the TVCs reported from the UBV sample alone in this case.

E.coli and Coliforms analysis results were negative in all the samples tested, indicating that the water was likely to be free of pathogens associated with faeces of both human and animal origin [32]. However, in all the six potable water tanks Pseudomonas was detected at levels ranging from 5 to 980 cfu/100ml for UBV samples and 420 to 3110 cfu/100ml for FE samples. According to HSE’s drinking water standards, the Pseudomonas count should be zero, or ‘non-detected’ [30]. It is likely that the presence of both biofilm and sedimentation towards the far end of the tanks (Table 2) was responsible [33, 34]. Pseudomonas aeruginosa is a bacterial strain, found widely in soil and stagnant water, and can infect humans and plants. It does not cause illness in healthy people, but can cause serious infections in immunosuppressed individuals. Infection of the lung may result in a form of pneumonia, extensive tissue damage may result from infected wounds or burns, and infection of the gastro-intestinal system may result in "necrotising enterocolitis" [35]. Some studies have confirmed that Pseudomonas growth in drinking water is probably related to its ability to colonize biofilms in plumbing fixtures [36,37]. Pseudomonas may be found in both low and high nutrient environments, including waste water and sewage water where it is reported to be associated with a wide range of infections in immunocompromised individuals [38,39].

The presence of Pseudomonas and associated biofilm is also a risk factor for other pathogenic bacteria, including *Legionella* [40]. This is illustrated here using the water sample analysis result from T13, where the TVC analysis and Pseudomonas counts were high in both cases, suggesting the a higher risk of *Legionella* proliferation. Analysis confirms the presence *Legionella* in both UBV and FE samples (200 cfu/L and 800cfu/L, respectively) of T13. Changes to the internal environment of the tank from the ball valve end to the far end of the tank are supported by visual inspection reports produced at the time of water sampling (Table 2) and additional photographic evidence included in this study (Figs 6 and 7). On the basis of the routine inspection report and the results presented, the increasing trend of microbial activity towards the far end of the tanks is likely due to increasing temperature, water stagnation, presence of biofilm and sedimentation with distance from the ball valve [41,42].

In this study, variations in water temperature from the mains supply end to far end of some tanks increase by as much as 3–3.6°C. Temperature is known to play an important role in the colonization of *Legionella* bacteria in water systems [43]. *Legionella* bacteria can survive and persist at temperatures between 6 and 63°C although proliferation is generally accepted to occur between 20–45°C and when suitable nutrients are available [44]. Recent studies, however, suggest that *Legionella* can replicate between 12–17°C, when other conditions favour their proliferation [45]. Although the temperature of the mains water entering the tank was at, or below, 12°C in the winter months, the temperature at the far end of the tanks exceeded 12°C in almost all cases, with the exception of two tanks sampled in the winter (December and January). Therefore, the temperature of the stored water at the far end of the tank may reach optimum levels for *Legionella* proliferation if the water is not frequently replenished [9]. Indeed, T11 and T13 both contained live *Legionella pneumophila* (100cfu/L and 800cfu/L, respectively), and had water temperatures at the far end that exceeded 20°C. According to Health Technical Memorandum Part B produced by UK Health Department in 2016, incoming mains water temperature can reach up to 25°C in the summer season [46] and the water temperature at the far end can reach well above 30°C; close to the maximum virulence temperature for *Legionella* bacteria [47]. In effect, there is a significant difference in the temperature of the stored water under the ball valve and at the far end of the tank which is influenced by seasonal variables (Fig 2), resulting in clear differences in the microbiological quality of water samples taken from these two locations. Water stagnation is also recognised to be a major factor in water hygiene maintenance and management [48]. A number of studies have confirmed that stagnant water provides ideal conditions for microbiological growth to occur [49]. For example, overnight stagnation of drinking water in household taps was found to be associated with a 2–3 fold increase in microbial concentrations and changes to the bacterial community composition. However, after flushing the taps for 5 minutes, bacteria concentrations and water temperatures decreased to levels generally found in the drinking water network [50, 51]. Visual observations of the water contained within the tanks invetsigated here also found evidence of surface water stagnation, due to a combination of slow outgoing of water from the bottom of the tank and poor internal water circulation [27]. Surface water stagnation is an important causal factor for biofilm formation, thereby creating a perfect breeding ground for pathogenic bacteria, including *Legionella* pneumophila [52].

Biofilms are known to be a major source of bacterial contamination, and are often responsible for recurrent contamination of water systems by *Legionella* pneumophila [53]. In natural environment, biofilms are typically described as complex, natural assemblages of various types of microorganism involved in a multitude of trophic and symbiotic interactions [54]. Although biofilms often typically start in nutrient rich environments (where bacteria change from free-living planktonic cells to sessile surface bound cells state), their presence represents a protected mode of growth allowing different types of cells to survive in hostile environments for extended periods of time, and also to disperse to colonize new niches when environmental conditions change [55–57]. Once established, biofilms can cause biocorrosion of water storage and supply materials, and are a major cause of disinfection inefficiency, serving as reservoirs for various pathogenic and non-pathogenic microorganisms, including *Legionella* [58]. Biofilm growth was consistently greater at the far end of the tank (Table 3). Indeed, only 25% of tanks surveyed had biofilms at the ball valve end and these were characterised as ‘slight’ and occurred in the spring and summer periods. In contrast, 90% of all tanks surveyed had biofilm at the far end of the tank (50% ‘slight’, 20% ‘Moderate’ and 21% ‘severe’) and these occurred throughout the year despite seasonal changes in incoming mans temperatures (Table 3). 11 out of the 15 tanks surveyed here were also noted to have surface water biofilms at the far end of the tank. Importantly, FE water samples had consistently higher levels of microbiological activity (TVCs and Pseudomonas counts). Therefore, analysis of water samples taken under the inlet ball valve (where samples are routinely taken for regulatory compliance) is not representative of the actual overall condition of the stored water due to biofilm growth associated with water stagnation.

**Fig 8:**
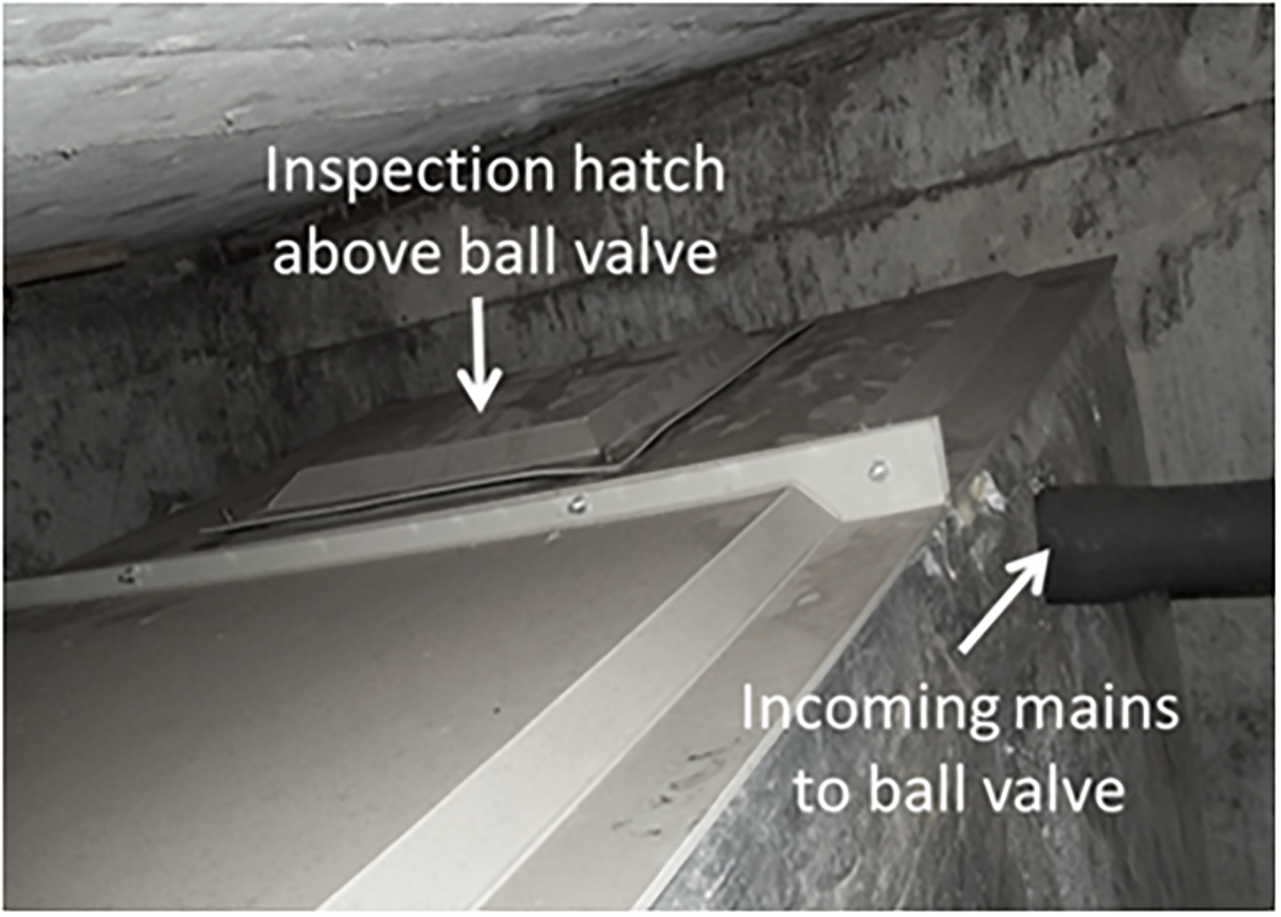
Position of inspection hatch and inlet on cold water storage tank. A typical 4 metre long metal cold water storage tank with internal butyl lining showing the position of the inspection hatch and mains inlet to the ball valve.

Tiny suspended and dissolved solids are carried in the mains water and settle in the bottom of water storage tanks to form sedimentary deposits [59]. Corrosion products, scale and sediments then act together as nutrients, encouraging *Legionella* proliferation [14]. According to a study carried out by Veterans Administration Medical Centre and University of Pittsburgh, the presence of sediment in stored water enhances the survival of *Legionella pneumophila* directly by acting as a nutrient, but also indirectly by encouraging the growth of other environmental bacteria that interact with Legionella via nutritional symbiosis. The bacteria and sediments act synergistically, in combination, to improve the survival of bacteria, including *Legionella pneumophila* [60]. Here we found that eleven of the tanks with microbiological activity also had sedimentation at the far end of the tanks, whereas the bottom of the tank under the inlet valve was comparatively free from sediments (Table 3). As with the biofilms, sedimentation became more severe with distance from the inlet valve, suggesting that a greater risk of bacterial contamination would occur from the mains inlet to the far end of the tank. The sample collected from the far end of the tank therefore more accurately represents the actual quality of the stored water entering the building system, and it is therefore vital to collect water samples from this location. Unfortunately, the the far end of most water storage tanks are sealed and completely innacessible. Current WRAS guidelines, state that the inspection and sampling access hatch location should be above the inlet ball valve (Figs 1 and 8) in order to facilitate maintenance of the inlet/ball valve [19].

### Public health perspective

**Fig 9:**
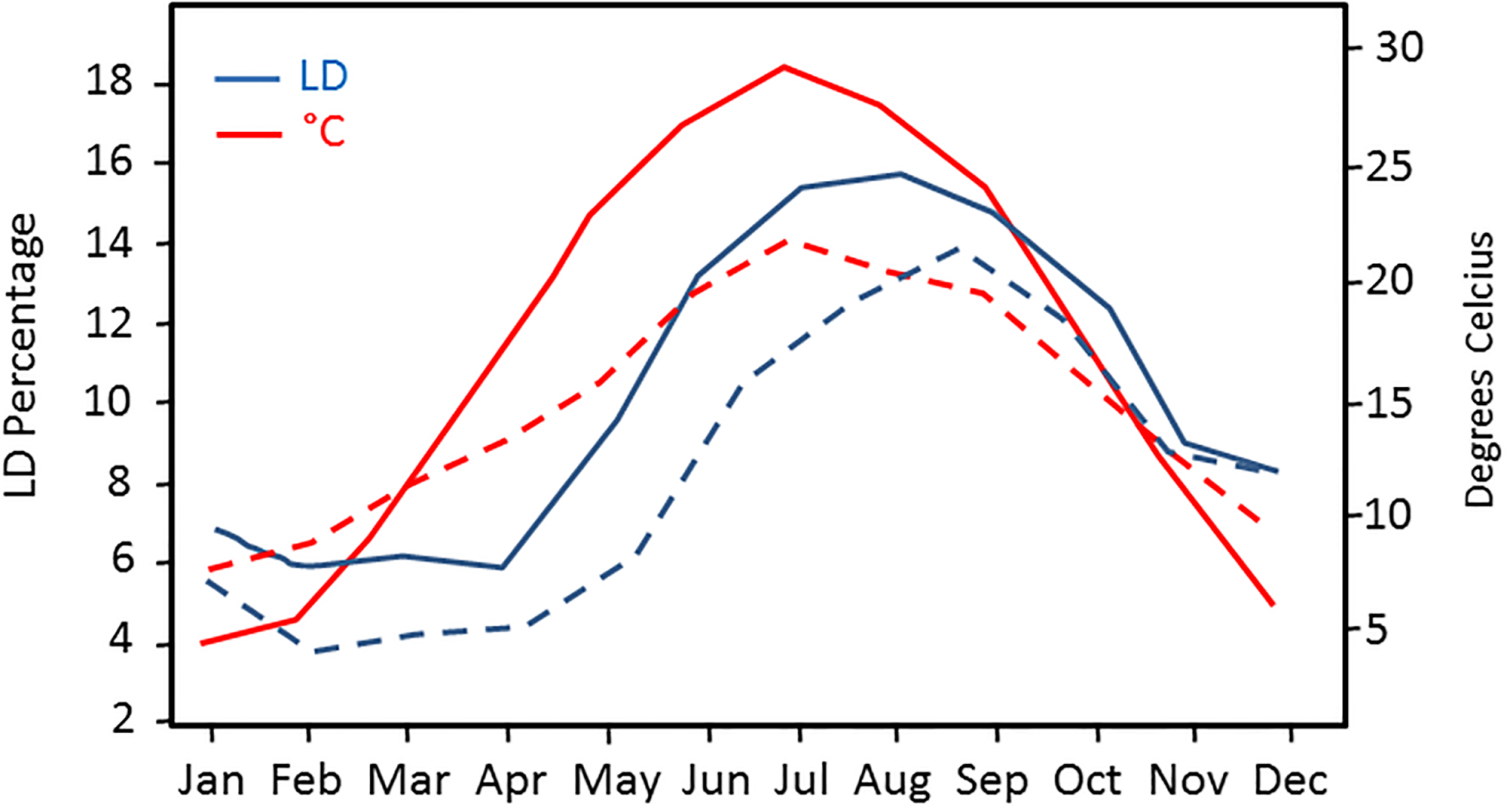
Comparison of LD cases in the USA and UK. Average percentage of LD cases occurring in the United States and UK annually by month. U.S. census data 2000–2009 and UK census data from 2015 and 2016 relative to seasonal high temperatures (District of Colombia and average UK temperature). Census data reported to Centers for Disease Prevention and Control (CDC) through the National Notifiable Disease Surveillance System (NNDSS) and a Supplemental Legionnaires Disease Surveillance System (SLDSS). UK data on Legionnaires disease was acquired from Public Health England reports. Solid line represents USA and dashed line is the UK.

Even in modern society, waterbourne pathogenic bacteria continue pose a serious threat to human health. Legionnaires’ disease (LD), caused by *Legionella pneumophila*, is just one of a number of potentially fatal diseases associated with water related infections [61]. Importantly, the internal conditions of the tanks used to store water, and associated water quality parameters, will influence the rate of proliferation of *Legionella* bacteria within the water system, the risk of exposure to contaminated aerosols containing *Legionella* bacteria during normal water usage, and the likelihood of contracting in LD [44]. Water temperatures between 20–45°C are known to encourage Legionella growth within water systems [62, 42] and studies in United States and Europe have confirmed that stored cold water temperature is likely to rise above 20°C in summer, consistent with greater numbers of community acquired Legionnaires disease [63, 64, 65]. We found that incoming mains water of ~ 20°C could reach 22–23°C at the FE of tanks greater than 1 metre in length in the summer. Legionella proliferation (if present) is likely to occur quickly in the warmer and nutrient rich water at the far end of the tank, where it is then abstracted for use in the building. The number of reported cases of Legionnaires’ disease in the USA follows a seasonal trend, being much higher during the summer season [63], and the seasonal prevalence of LD appears to be worsening, possibly as a result of climate change [64]. Indeed, the number of reported LD cases in The Netherlands was unusually high in the summer of 2010, associated with warmer and wetter climatic conditions [65]. Furthermore, the outcome of these studies agree with official statistics on LD published by both US and UK government agencies (Fig 9). Given the disparity between measurements taken at different end of the tanks, we propose that monitoring at the far end of cold water storage tanks would provide a more accurate and relevant indication of microbiological contamination, enabling appropriate precautions to be taken to protect the public from water bourne pathogenic diseases, including Legionnaires’ disease.

## Recommendations and conclusions

Exposure to pathogenic bacteria in buildings is a known public health risk, and the strategies used to protect society from exposure to all pathogenic bacteria must be constantly reviewed and revised [66]. Cold water storage tanks are an important source of repeated bacterial contamination in buildings, resulting in risks of exposure to the building users. Consequently, regulations require that a sample of tank water is taken for regular microbiological monitoring as part of the risk management strategy to control *Legionella* and other pathogenic bacteria. It is generally assumed that the water sample will provide a representative view of the microbiological status of the entire tank in order to inform risk management stategies. Here we report large differences in the microbiological status of water samples collected under the ball valve (where there is easy access, and where samples are routinely taken for regulatory compliance) compared to the far end of the tank where water typically enters the building but where sampling access is constrained. Water samples collected under the ball valve and analysed by an accredited laboratory were found to have almost identical characteristics to the incoming mains, and were not representative of the stored water at the far end of the tank. In order to control *Legionella* and maintain water hygiene standards, it is vital that a representative sample of stored water entering the building is collected as part of routine monitoring.

According to our results, and withfew exceptions, water temperature, the level of sedimentation and biofilm (known risk factors for the establishment of Legionella and other water bourne pathogens) increased in quantity/severity between UBV and FE in both cold water and potable water storage tanks. Consequently, 20% of the tanks surveyed failed to trigger appropriate regulatory action based on microbiological analyses of the water sample taken under the ball valve compared to the far end sample using present-day standards. These results call into question the reliability of present measures used to protect the public from water bourne pathogenic diseases, including Legionella.

In general, the smaller tanks (≤ 1 m^3^) investigated in this study showed greater consistency in water quality parameters (including the presence of biofilm, sedimentation level, bacterial concentration and temperature). However, large disparities in water quality parameters used to protect the public from exposure to pathogenic bacteria were noted in larger tanks greater than one metre in length. In order to comply with current WRAS guidelines, any cold water storage tank of more than 1000 litres capacity should have a screened warning pipe and a screened overflow [67]. In the same way, we propose that new water storage tanks of similar capacity should be fitted with an additional inspection and sampling access hatch at the far end of the tank, and this requirement could be imposed through appropriate national and international guidelines.

## Supporting information

S1 Table Raw data. Water sample temperatures and analysis results (TVC, E. Coli, Coliforms, Pseudomonas and Legionella pneumophila) used to generate the Figs 2–5. IM—incoming mains; UB—under the ball valve; FE—far end.
(PDF)
