# Supplementary material for: Present-day monitoring underestimates the risk of exposure to pathogenic bacteria from cold water storage tanks
Source: PLoS One. 2018 Apr 12;13(4):e0195635. doi: 10.1371/journal.pone.0195635 (PMC5896965; doi:10.1371/journal.pone.0195635)
Supplement: S1 Table — Water sample temperatures and analysis results (TVC, E. Coli, Coliforms, Pseudomonas and Legionella pneumophila) used to generate the Figs 2–5. IM—incoming mains; UB—under the ball valve; FE—far end. (PDF) [file pone.0195635.s001.pdf]

**S1 Table. Raw data.** Water sample temperatures and analysis results (TVC, E. Coli, Coliforms, Pseudomonas and Legionella pneumophila) used to generate the figures 2-5. IM – incoming mains; UB – under the ball valve; FE – far end.

| Sample Ref. | Sample temperature | TVC(3 days @ 22˚C ) cfu/100 ml | TVC (2 days @ 37˚C) cfu/100ml | Pseudomonas spp (cfu/100ml) | E.Coli | Coliforms | Legionella Spp cfu/L |
|---|---|---|---|---|---|---|---|
| **T1 - IM** | 12.5 | 0 | 0 | 0 | 0 | 0 | 0 |
| **T1 - UB** | 12.5 | 0 | 0 | 0 | 0 | 0 | 0 |
| **T1 - FE** | 12.6 | 38 | 0 | 1000 | 0 | 0 | 0 |
| **T2 - IM** | 12.5 | 0 | 0 | 0 | 0 | 0 | 0 |
| **T2 - UB** | 12.5 | 0 | 0 | 0 | 0 | 0 | 0 |
| **T2 - FE** | 12.5 | 3000 | 23 | 0 | 0 | 0 | 0 |
| **T3 - IM** | 7 | 0 | 0 | 0 | 0 | 0 | 0 |
| **T3 - UB** | 8 | 0 | 0 | 5 | 0 | 0 | 0 |
| **T3 - FE** | 9 | 1100 | 0 | 500 | 0 | 0 | 0 |
| **T4 - IM** | 7 | 0 | 0 | 0 | 0 | 0 | 0 |
| **T4 - UB** | 7 | 9 | 0 | 3 | 0 | 0 | 0 |
| **T4 - FE** | 10 | 1300 | 85 | 27 | 0 | 0 | 0 |
| **T5 - IM** | 9.3 | 0 | 0 | 0 | 0 | 0 | 0 |
| **T5 - UB** | 9.8 | 97 | 6 | 12 | 0 | 0 | 0 |
| **T5 - FE** | 13.2 | 4200 | 165 | 1100 | 0 | 0 | 0 |
| **T6 - IM** | 8.4 | 1 | 0 | 0 | 0 | 0 | 0 |
| **T6 - UB** | 8.4 | 56 | 2 | 9 | 0 | 0 | 0 |
| **T6 - FE** | 12 | 2300 | 130 | 760 | 0 | 0 | 0 |
| **T7 - IM** | 8.5 | 0 | 0 | 0 | 0 | 0 | 0 |
| **T7 - UB** | 8.7 | 67 | 1 | 0 | 0 | 0 | 0 |
| **T7 - FE** | 13 | 1400 | 185 | 1390 | 0 | 0 | 0 |
| **T8 - IM** | 12.7 | 0 | 0 | 0 | 0 | 0 | 0 |
| **T8 - UB** | 12.9 | 50 | 0 | 7 | 0 | 0 | 0 |
| **T8 - FE** | 14.8 | 2900 | 170 | 500 | 0 | 0 | 0 |
| **T9 - IM** | 13.1 | 2 | 0 | 0 | 0 | 0 | 0 |
| **T9 - UB** | 13.3 | 64 | 13 | 17 | 0 | 0 | 0 |
| **T9 - FE** | 15.2 | 2500 | 430 | 960 | 0 | 0 | 0 |
| **T10 - IM** | 16.4 | 0 | 0 | 0 | 0 | 0 | 0 |
| **T10 - UB** | 16.7 | 1974 | 1140 | 83 | 0 | 0 | 0 |
| **T10 - FE** | 18.0 | 2362 | 1900 | 680 | 0 | 0 | 0 |
| **T11 - IM** | 17.7 | 0 | 0 | 18 | 0 | 0 | 0 |
| **T11 - UB** | 18.9 | 3700 | 2620 | 980 | 0 | 0 | 0 |
| **T11 - FE** | 20.2 | 5930 | 3170 | 3110 | 0 | 0 | 100 |
| **T12 - IM** | 18.4 | 12 | 0 | 0 | 0 | 0 | 0 |
| **T12 - UB** | 18.5 | 560 | 120 | 9 | 0 | 0 | 0 |
| **T12 - FE** | 19.1 | 2300 | 900 | 420 | 0 | 0 | 0 |
| **T13 - IM** | 19.0 | 10 | 0 | 9 | 0 | 0 | 0 |
| **T13 - UB** | 19.2 | 1670 | 840 | 290 | 0 | 0 | 200 |
| **T13 - FE** | 20.7 | 5400 | 3850 | 2200 | 0 | 0 | 800 |
| **T14 - IM** | 17.9 | 0 | 0 | 0 | 0 | 0 | 0 |
| **T14 - UB** | 19.2 | 169 | 1 | 5 | 0 | 0 | 0 |
| **T14 - FE** | 19.8 | 1400 | 285 | 490 | 0 | 0 | 0 |
| **T15 - IM** | 17.0 | 0 | 0 | 0 | 0 | 0 | 0 |
| **T15 - UB** | 17.0 | 10 | 1 | 0 | 0 | 0 | 0 |
| **T15 - FE** | 17.0 | 10 | 2 | 0 | 0 | 0 | 0 |
